# Extract of *Litsea japonica* (Thunba.) Jussieu Fruit Reduces Muscle Atrophy in Cancer Cachexia

**DOI:** 10.1002/fsn3.70624

**Published:** 2025-07-16

**Authors:** Sang Woo Woo, Pooreum Lim, Jihye Han, Ho Jin Kang, Jae Ho Shim, Jin Ju Lim, Seon‐A Yoon, Hyejin Hyeon, Young‐Min Ham, Ji‐Gweon Park, Yong‐Hwan Jung, Hyeon Soo Kim

**Affiliations:** ^1^ Department of Anatomy Korea University College of Medicine Seoul Korea; ^2^ Biodiversity Research Institute, Clean Bio Business Division, Jeju Technopark Jeju Korea

**Keywords:** cancer cachexia, *Litsea japonica* fruit, muscle atrophy, myostatin, skeletal muscle

## Abstract

Cancer cachexia is a complex syndrome involved in muscle wasting, fat depletion, fatigue, reduced appetite, and unintentional weight loss. Recent studies have suggested that natural products can prevent cancer cachexia; however, there have been no studies on the effects of *Litsea japonica* fruit extract on cancer cachexia. This study aimed to identify compounds of *L. japonica* fruit extracted from water (LJFE‐W) and those extracted from 30% ethanol (LJFE‐E) for cancer cachexia treatment. In vitro and in vivo models were used for C26 conditioned medium (CM)‐induced C2C12 myotubes and C26 tumor‐bearing mice. We demonstrated that LJFE‐W and LJFE‐E regulated myostatin (MSTN), E3 ligase muscle‐specific RING finger protein‐1 (MuRF1), and muscular atrophy fbox‐1 protein (MAFbx32) expression in a CM‐induced in vitro model. LJFE‐E ameliorated conditioned medium‐induced myotube atrophy in cultured C2C12 myotubes. In contrast, LJFE‐W and LJFE‐E stimulated the Akt–mTOR signaling pathway for protein synthesis in C2C12 myotubes. In animal models, the LJFE‐E‐injected C26 tumor‐bearing group showed lower transcript‐level expression of MSTN, MuRF1, and MAFbx32 in the gastrocnemius muscles than the C26 tumor‐bearing group. LJFE ameliorated muscle atrophy in the cancer cachexia model by inhibiting MSTN. Thus, LJFE can be used as a supplement to cancer cachexia therapy.

## Graphical abstract

This study demonstrated that Litsea japonica fruit extract (LJFE), especially LJFE‐E (30% ethanol extract), alleviated muscle atrophy by modulating myostatin (MSTN) in vitro and in vivo cancer cachexia models. These findings highlight LJFE as a promising supplement for cancer cachexia.

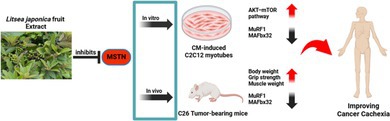

**Abbreviations**
Akt: protein kinase B
CM: C26‐conditioned medium
FOXO3a: forkhead box O3
IGF1: insulin‐like growth factor‐1
MAFbx32: muscular atrophy fbox‐1 protein
MSTN: myostatin
mTOR: mammalian target of rapamycin
MuRF1: E3 ligase muscle‐specific RING finger protein‐1
SMAD: suppressor of Mothers against Decapentaplegic

## 1 Introduction

Cancer cachexia is a multifactorial syndrome characterized by muscle atrophy, fat loss, fatigue, anorexia, and involuntary weight loss. Diagnostic criteria for cancer cachexia include a body mass index (BMI) or body weight loss and sarcopenia (Fearon et al. 2011). Cancer cachexia affects a significant proportion of cancer patients and contributes substantially to cancer‐related mortality (Thibaut et al. 2021). Cachexia is acknowledged as a major negative consequence of cancer progression, leading to diminished physical function, reduced tolerance to anticancer treatments, and poor survival rates (Neyroud et al. 2021). The pathogenesis of cancer cachexia encompasses metabolic disorders, chronic inflammation, and the dysregulation of skeletal muscle protein homeostasis (Dolan et al. 2019; Schwartsburd 2019).

Protein homeostasis, particularly the balance between protein synthesis and degradation, plays a central role in cancer cachexia development. Inflammatory cytokines released by tumors activate muscle atrophy factors, such as the E3 ligase muscle‐specific RING finger protein‐1 (MuRF1) and muscular atrophy F‐box protein‐1 (MAFbx32) and increase cell apoptosis. In addition, inflammatory cytokines and oxidative stress impair muscle protein synthesis pathways, notably the Akt–mTOR pathway (Setiawan et al. 2023).

Myostatin (MSTN), a negative regulator of muscle mass, plays a crucial role in maintaining skeletal muscle homeostasis by inhibiting muscle growth. MSTN binds to the activin type IIB receptor (ActRIIB) on muscle cell membranes, leading to activation of SMAD2 and SMAD3. Phosphorylated SMADs promote dephosphorylation of the transcription factor FOXO3a, which subsequently translocates into the nucleus to activate transcription of the E3 ubiquitin ligases MuRF1 and MAFbx32 (Farhang‐Sardroodi and Wilkie 2020; Bataille et al. 2021). Myostatin also inhibits the insulin/IGF1–AKT–mTOR pathway, thereby suppressing protein synthesis (Amirouche et al. 2009; Schiaffino et al. 2013).

Natural products have been valued for centuries as therapeutic resources for various diseases and physical ailments, offering therapeutic benefits with minimal adverse effects in humans (Yadav et al. 2022). These natural products are inexpensive, widely available, low in toxicity, and offer a potential novel approach to alleviating certain underlying symptoms of disease (Bagherniya et al. 2022). Natural products, such as terpenoids (Liu et al. 2019), polyphenols (Nikawa et al. 2021), and flavonoids (Hoek‐van den Hil et al. 2015), are widely known to reduce oxidative stress and inflammation. Additionally, various natural products have been reported to play prominent roles in the prevention and treatment of muscle‐related diseases (Qu et al. 2021; Han et al. 2023).

Litsea japonica (Thunb.) Jussieu is an evergreen tree native to southern Korea and Japan. This plant and its fruits are consumed in Korea, are rich in essential oils, fatty acids, lactones, alkaloids, and terpenoids (Jeong et al. 2015). Previous studies have reported that these fruits exert anti‐inflammatory and antioxidant effects (Yoon et al. 2010; Kim et al. 2017). However, the muscle atrophy effects of *L. japonica* fruit have not been thoroughly investigated.

This study examined whether water and ethanol extracts of *L. japonica* fruit could improve cancer cachexia, focusing on the effects of the extracts on MSTN activity using both in vivo and in vitro models. This study provides new insights into the anti‐cachexia potential of *L. japonica* fruit extracts.

## 2 Materials and Methods

### 2.1 Preparation of LJFE

The fruits utilized in this study were sourced from Jeju Island in Korea, with assistance from the Jeju Biodiversity Research Institute, supported by Jeju Technopark. A voucher specimen (No. JBRI‐079) has been deposited at the Jeju Biodiversity Research Institute. The dried fruit (1000 g) was subjected to two types of extractions, first with water (LJFE‐W) and then with 30% ethanol (LJFE‐E), each for 18 h at 60°C. The resulting extracts were concentrated under reduced pressure. The decoction was subsequently filtered and lyophilized to produce the LJFEs.

### 2.2 Antibodies

The primary antibodies against rabbit MSTN (#GTX32624) were purchased from Gentex. Primary antibodies against rabbit anti‐MuRF1 (#ab172479) and rabbit anti‐MAFbx32 (#ab168372) were purchased from Abcam. Primary antibodies against rabbit anti‐p‐AKT (Ser473) (#4060), rabbit anti‐AKT (#9272), rabbit anti‐p‐mTOR (Ser2448) (#2971S) and rabbit anti‐p‐mTOR (#2972) were purchased from Cell Signaling Technology. Primary antibodies against mouse β‐actin (#E12‐041‐4) were purchased from Enogene. Secondary horseradish peroxidase (HRP)‐conjugated anti‐mouse (#ADI‐SAB‐100‐J) and anti‐rabbit (#ADI‐SAB‐300‐J) were purchased from ENZO.

### 2.3 Cell Culture

C2C12 myoblasts (ATCC) were cultured in proliferation medium composed of Dulbecco's modified Eagle's medium (DMEM) supplemented with 10% fetal bovine serum (Gibco) and 1% penicillin and streptomycin (Welgene) at 37°C in a humidified incubator with an atmosphere of 5% CO2. To induce myogenic differentiation, when the C2C12 myoblasts reached 90% confluence, the proliferation medium was replaced with a differentiation medium consisting of DMEM supplemented with 2% horse serum (Gibco) starting from Day 0. The differentiation medium was changed daily, and myotubes were fully differentiated by Day 5, at which point the experiments were performed. The C26 mouse colon adenocarcinoma cell line was maintained in proliferation medium consisting of Roswell Park Memorial Institute 1640 (RPMI1640, Welgene) cultured with 10% fetal bovine serum (Gibco) and 1% penicillin and streptomycin (Welgene) at 37°C in a humidified incubator with 5% CO2.

### 2.4 Collection of Conditioned Medium (CM)

To collect CM, C26 cells were cultured at a density of 5 × 10^6^ cells in 10 cm culture plates. After overnight attachment, cells were washed twice with PBS and cultured in serum‐free DMEM for 48 h. The resulting CM was centrifuged at 500 × *g* for 10 min and then at 3000 × *g* for 10 min. The CM was filtered using a 0.2 μm syringe filter and either immediately used or stored at −80°C. CM was used immediately at a 1:5 dilution in fresh normal medium.

### 2.5 Myostatin Luciferase Reporter Assay

Luciferase reporters were utilized to evaluate the activity of the MSTN promoter in C2C12‐MSTN myoblast cells, prepared as described (Kang et al. 2022). Stably transfected C2C12‐MSTN cells (2 × 10^5^) were seeded into 12‐well plates. To determine whether MSTN promoter activity was inhibited, myoblasts were treated with various compounds for 12 h. Luciferase activity was assessed using the BrightGlo Luciferase Assay System (Promega) following the manufacturer's protocol. All experiments were conducted and validated at least three times.

### 2.6 MTT Assay

C2C12 myoblasts (2 × 10^4^ cells/well) were seeded in 96‐well plates. After the cells adhered, they were treated with 100 μL fresh growth medium containing various concentrations of LJFE‐W and LJFE‐E for 24–72 h. Then, 10 μL of MTT (5 mg/mL) was added to the medium at 37°C for 4 h. After discarding the medium, formazan crystals were measured at a wavelength of 540 nm.

### 2.7 Real‐Time PCR

Total RNA was extracted from C2C12 myotubes and freeze‐dried mouse skeletal muscles using QIAzol (Qiagen, Hilden, Germany). The RNA concentration was measured using a NanoDrop spectrophotometer (Thermo Fisher Scientific). Complementary DNA (cDNA) was synthesized from 5 μg RNA using a reverse transcription system (Promega). The expression of mRNA was assessed by real‐time PCR (qRT‐PCR) (QuantStudio 3 Real‐Time PCR, Thermo Fisher Scientific) using a SYBR Green system (TOPreal qPCR 2X PreMIX, Enzynomics). The following mouse primers were used: MSTN forward (5‐GCA CTG GTA TTT GGC AGA GT‐3), MSTN reverse (5‐TTC AGC CCA TCT TCT CCT GG‐3), GAPDH forward (5‐GTG TTC CTA CCC CCA ATG TG‐3), GAPDH reverse (5‐CCT GCT TCA CCA CCT TCT TG‐3), MuRF1 forward (5‐GTC CAT GTC TGG AGG TCG TT‐3), MuRF1 reverse (5‐AGG AGC AAG TAG GCA CCT CA‐3), MAFbx32 forward (5‐ATG CAC ACT GGT GCA AAG AG‐3), MAFbx32 reverse (5‐TGT AAG CAC ACA GGC AGG TC‐3), MHC1 forward (5‐TGC AGC AGT TCT TCA ACC AC‐3), MHC1 reverse (5‐TCG AGG CTT CTG GAA GTT GT‐3), MHC2a forward (5‐AGT CCC AGG TCA ACA AGC TG‐3), MHC2a reverse (5‐GCA TGA CCA AAG GTT TCA CA‐3), MHC2b forward (5‐AGT CCC AGG TCA ACA AGC TG‐3), MHC2b reverse (5‐TTT CTC CTG TCA CCT CTC AAC A‐3), MHC2x forward (5‐AGT CCC AGG TCA ACA AGC TG‐3), and MHC2x reverse (5‐CAC ATT TGG CTC ATC TCT TGG‐3). Data from each sample were normalized to GAPDH, and fold‐change values were calculated using the ΔΔCt method.

### 2.8 Western Blotting

C2C12 myotubes and tibialis anterior muscle were lysed in a lysis buffer containing protease and phosphatase inhibitors (GenDEPOT). The lysates were loaded onto 10% SDS‐PAGE gels and transferred onto 0.45 μm nitrocellulose membranes (GE Healthcare). The membranes were blocked in Tris‐buffered saline with Triton X‐100 (TBST) containing 0.1% Tween 20 and 5% dry milk (w/v) for 1 h and then washed with TBST. Membranes were incubated overnight with the following primary antibodies and then probed with appropriate HRP‐conjugated secondary antibodies for 1 h. Chemiluminescence on the blots was visualized using the Amersham Biosciences ECL Detection System (GE Healthcare).

### 2.9 Animal Experiments

Animal experiments were approved by the Korea University Institutional Animal Care and Use Committee and performed in accordance with relevant guidelines and regulations (KOREA‐2021‐0142‐C2). Male BALB/C mice (*n* = 48) 8 weeks of age were obtained from Japan SLC (Hamamatsu, Japan) and housed in a highly controlled environment (temperature, 21°C–23°C; relative moisture, 50%–60%; 12 h light:12 h dark cycle). Mice had access to water *ad libitum* and were fed a standard chow diet. All animals were acclimated for 1 week before beginning the study and then divided randomly into four groups (*n* = 12 per group): normal, C26 tumor‐bearing, C26 tumor‐bearing treated with LJFE‐W (100 mg/kg), and C26 tumor‐bearing treated with LJFE‐E (100 mg/kg). C26 tumor‐bearing mice were subcutaneously injected with c26 cells (1 × 10^6^ cells/100 μL) on the flanks of both sides. One week after C26 injection, the mice received either an IP injection of LJFE‐W, LJFE‐E (100 mg/kg in saline), or saline (vehicle) daily for 3 weeks. Mice were carefully monitored, and body weight, food intake, and water intake were measured daily from the time of C26 cell inoculation until the end of the experiment. If mice lost 20% or more of their body weight rapidly within a short period, the experiment was halted, and the mice were euthanized. After 48 h after the last behavioral tests, blood was collected after a 12‐h fast, and the tumor and hindlimb muscles were excised under isoflurane anesthesia for analysis. Wide resections were performed on hind limb muscle tissues, which were then washed with phosphate‐buffered saline.

### 2.10 Grip Strength Test

To assess grip strength of the forelimbs (two mouse paws), a grip strength test was conducted using a calibrated grip strength tester (Jeung Do Bio & Plant Co., JD‐A‐22). The results of grip strength analysis are presented as the mean of at least three repeated measurements. Grip strength tests were conducted weekly.

### 2.11 Histology

Tibialis anterior muscles were fixed with formaldehyde (4%), dehydrated in a graded ethanol series, and cleared in xylene using a Leica AS300S tissue processor (Leica Microsystems). Paraffin‐embedded blocks were cut into 8 μm sections using a Leica rotary microtome (RM2255, Leica Microsystems), and the tissues were mounted on slides. Histological examination was performed by H&E staining. The stained sections were scanned using a slide scanner (Axio Scan Z1). To measure the myofibers cross‐sectional area, eight non‐overlapping areas of each section were digitally captured, and > 250 myofibers were calculated for each sample (*n* = 5). All muscle fiber areas were calculated using ImageJ (National Institute of Health).

### 2.12 Measurement of Inflammatory Cytokines

The mice blood samples were kept in anti‐coagulation tubes and pro‐coagulation tubes and then centrifuged (1000 *g*, 10 min, 4°C) to obtain platelet‐free plasma and serum, within 2 h after blood collection. Serum IL‐6 and TNF‐α were detected using an enzyme‐linked immunosorbent assay (ELISA) kit (R&D Systems, Minneapolis, MN, USA) according to the manufacturer's instructions.

### 2.13 Statistical Analysis

Data are expressed as mean ± standard error of the mean (SEM). Statistical differences between and among groups were evaluated using Student's *t*‐test and ANOVA, followed by Tukey's post hoc test. Prism 9 (GraphPad) software was used for statistical analyses, and *p* values < 0.05 were considered significant.

## 3 Results

### 3.1 LJFE Suppressed the Promotor Activity of MSTN in C2C12 Cells

**FIGURE 1:**
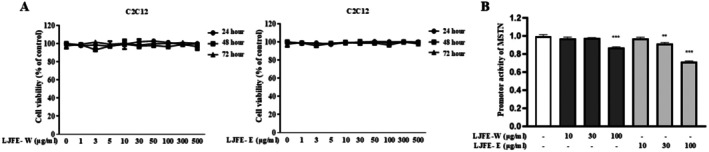
Cytotoxicity and myostatin inhibitory effect of LJFE. (A) Cell viability was determined using an MTT assay. C2C12 cells were treated with LJFE‐W or LJFE‐E at a concentration range of 0–500 μg/mL for 24–72 h. (B) Luciferase activity in stable myostatin C2C12 cells treated with LJFE‐W or LJFE‐E at the indicated doses (10, 30, and 100 μg/mL) for 12 h was assessed using a luciferase assay. Data points represent the mean ± standard error of the mean (SEM) of triplicate measurements. Statistical analysis was performed using one‐way ANOVA. *p < 0.05, **p < 0.01, and ***p < 0.001.

Before examining the effect of LJFE on C2C12 cells, its cytotoxicity was confirmed using MTT assay. C2C12 cells were treated with increasing concentrations (0–500 μg/mL) of LJFE‐W or LJFE‐E for 24–72 h. LJFE was not cytotoxic to C2C12 cells at a concentration of 500 μg/mL (Figure 1A). This study examined the effect of an optimal dose of LJFE on MSTN, a well‐known negative regulator of muscle atrophy, and used a stable luciferase reporter cell line with the MSTN promoter to determine whether LJFE‐W or LJFE‐E regulated MSTN via transcriptional regulation. It was found that LJFE inhibited MSTN promoter activity at 100 μg/mL LJFE‐W and at 30 μg/mL LJFE‐E (Figure 1B). These findings suggest that LJFE affects myostatin levels.

### 3.2 LJFE Inhibits MSTN Expression in CM‐Induced C2C12 Myotubes

**FIGURE 2:**
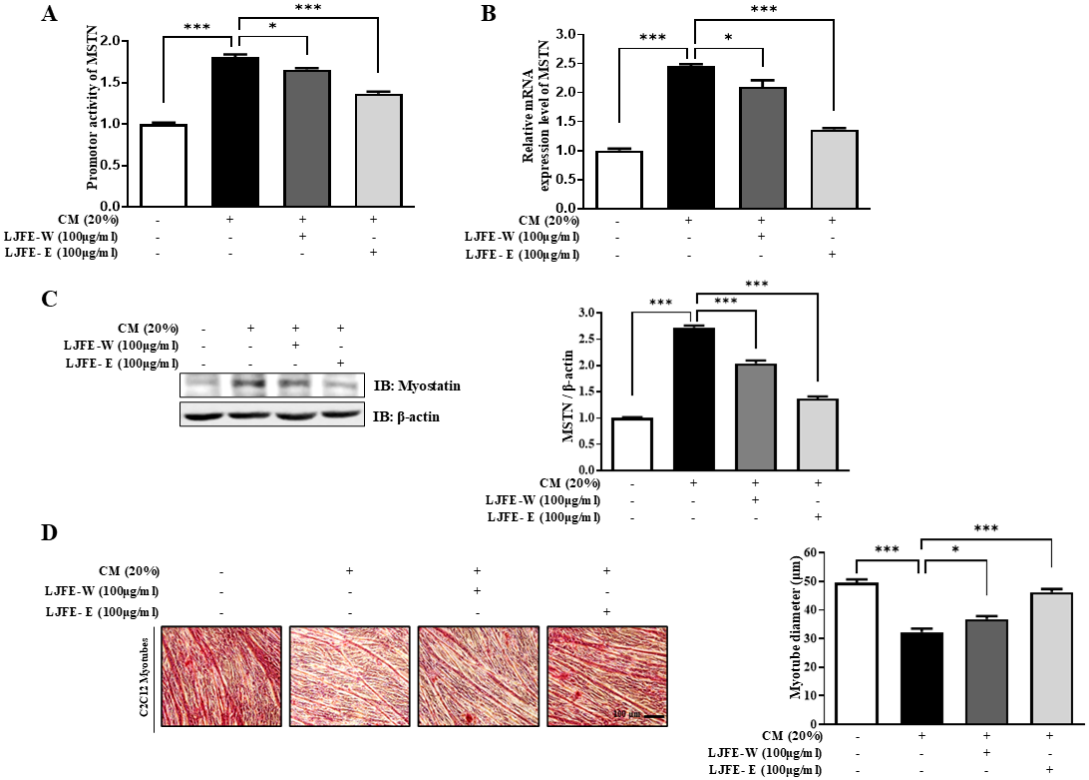
Inhibitory effect of LJFE on myostatin in CM‐induced muscle atrophy. (A) Luciferase activity in stable myostatin C2C12 cells cultured in DM containing CM 20% for 12 h with or without the indicated doses (100 μg/mL) of LJFE‐W or LJFE‐E for 12 h was assessed using a luciferase assay. (B) Comparison of the relative mRNA expression of myostatin using qPCR. C2C12 myotubes were co‐treated with LJFE‐W or LJFE‐E (100 μg/mL) and incubated with CM (20%) for 24 h. PCR was normalized to GAPDH. Bars represent the mean ± SEM. (C) The expression level of MSTN was evaluated by western blotting. C2C12 myotubes were co‐treated with LJFE‐W or LJFE‐E (100 μg/mL) and incubated with CM (20%) for 48 h. (D) Images of myotube morphology and analysis of diameter in C2C12 myotubes in DM and CM (20%) with or without indicated doses (100 μg/mL) of LJFE‐W or LJFE‐E for 48 h. Analysis of the diameter of cultured myotubes was calculated using Image J. Bars represent the mean ± SEM. Statistical analysis was performed using one‐way ANOVA. *p < 0.05, **p < 0.01, and ***p < 0.001.

To confirm that LJFE attenuated muscle atrophy, CM was prepared from cultured C26 colon carcinoma cells. Although CM increased MSTN promoter activity, LJFE‐W and LJFE‐E decreased MSTN levels (Figure 2A). MSTN expression in the CM‐treated cells was examined following LJFE treatment. 100 μg/mL of LJFE dramatically downregulated MSTN mRNA expression in CM‐induced C2C12 myotubes (Figure 2B). The protein levels of MSTN were confirmed by western blotting, and LJFE‐W and LJFE‐E substantially decreased CM‐induced MSTN expression (Figure 2C). Morphological analysis showed that C2C12 myotubes were fully differentiated, co‐treated with CM and LJFE‐W or LJFE‐E for 48 h. CM reduced the diameter of C2C12 myotubes; however, co‐treatment with LJFE counteracted this reduction (Figure 2D). These results suggest that LJFE exerts beneficial effects on MSTN levels in CM.

### 3.3 LJFE Inhibits Expression of Muscle Atrophy Markers in CM‐Induced C2C12 Myotubes

**FIGURE 3:**
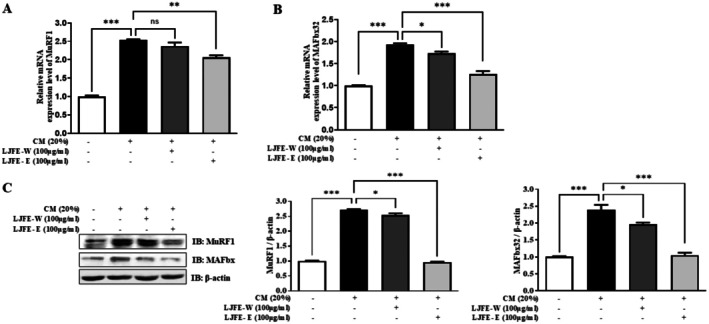
Ameliorating effects of LJFE on muscle atrophy markers in C2C12 myotubes induced by CM. (A) Comparison of the relative mRNA expression of MuRF1 using qPCR. C2C12 myotubes were treated with LJFE‐W or LJFE‐E (100 μg/mL) and incubated with CM (20%) for 24 h. PCR was normalized to GAPDH. Bars represent the mean ± SEM. (B) Comparison of the relative mRNA expression of MAFbx32 using qPCR. C2C12 myotubes were treated with LJFE‐W or LJFE‐E (100 μg/mL) and incubated with CM (20%) for 24 h. PCR was normalized to GAPDH. Bars represent the mean ± SEM. (C) The expression levels of MuRF1 and MAFbx32 were evaluated by western blotting. C2C12 myotubes were treated with LJFE‐W or LJFE‐E (100 μg/mL) and incubated with CM (20%) for 48 h. Statistical analysis was performed using one‐way ANOVA. *p < 0.05, **p < 0.01, and ***p < 0.001.

After confirming the effect of LJFE on MSTN CM models, we analyzed whether it had similar effects on the muscle atrophy markers MuRF1 and MAFbx32. LJFE‐E downregulated MuRF1 and MAFbx32 mRNA expression in CM‐treated C2C12 myotubes. However, LJFE‐W treatment did not significantly downregulate the mRNA expression of MuRF1 (Figure 3A,B). The protein levels of MuRF1 and MAFbx32 were measured using western blotting, which confirmed that they showed similar mRNA expression patterns (Figure 3C). These results indicate that LJFE‐E has more beneficial effects on muscle atrophy than LJFE‐W.

### 3.4 LJFE Stimulates the Akt–mTOR Protein Synthesis Signaling Pathway

**FIGURE 4:**
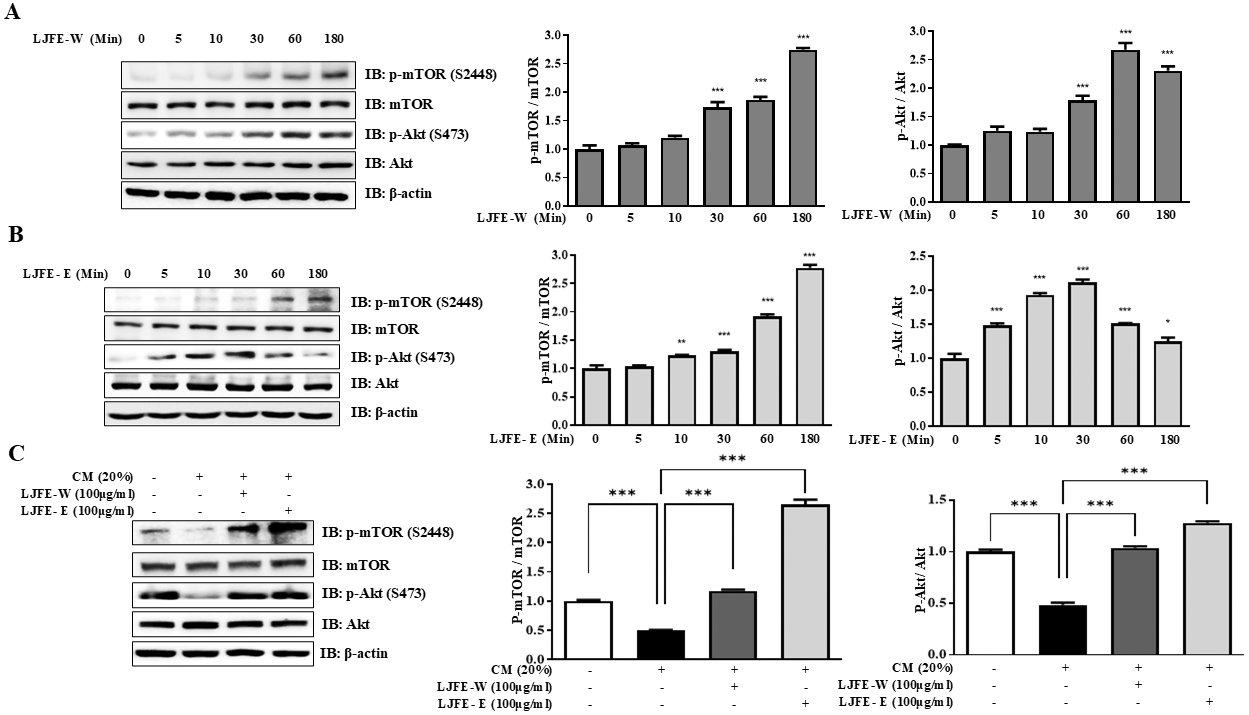
The effects of LJFE on muscle protein synthesis in C2C12 myotubes. (A) The expression levels of p‐mTOR (S2448), mTOR, p‐Akt (S473), and Akt were evaluated by western blotting. C2C12 myotubes incubated with or without LJFE‐W (0–100 μM) for time dependent manner. (B) The expression levels of p‐mTOR (S2448), mTOR, p‐Akt (S473), and Akt were evaluated by western blotting. C2C12 myotubes incubated with or without LJFE‐W (0–100 μM) for time dependent manner. (C) The expression levels of p‐Akt (S473), Akt, p‐mTOR (S2448), and mTOR were evaluated by western blotting. C2C12 myotubes were treated with LJFE‐W or LJFE‐E (100 μg/mL) and incubated with CM (20%) for 48 h.

This study examined the effects of LJFE on the Akt–mTOR pathway in a time‐dependent manner. Protein levels increased in the LJFE‐W and LJFE‐E groups (Figure 4A,B). We confirmed the effects of LJFE‐mediated Akt–mTOR regulation in CM. C2C12 myotubes were differentiated and co‐treated with CM and LJFE‐W or LJFE‐E for 48 h. CM decreased p‐Akt and p‐mTOR, with LJFE‐W or LJFE‐E preventing this (Figure 4C). Therefore, LJFE increased muscle protein synthesis by regulating the Akt–mTOR pathway in CM‐induced muscle atrophy.

### 3.5 LJFE Inhibits Muscle Atrophy in C26 Tumor‐Bearing Cancer Cachexia Mice

**FIGURE 5:**
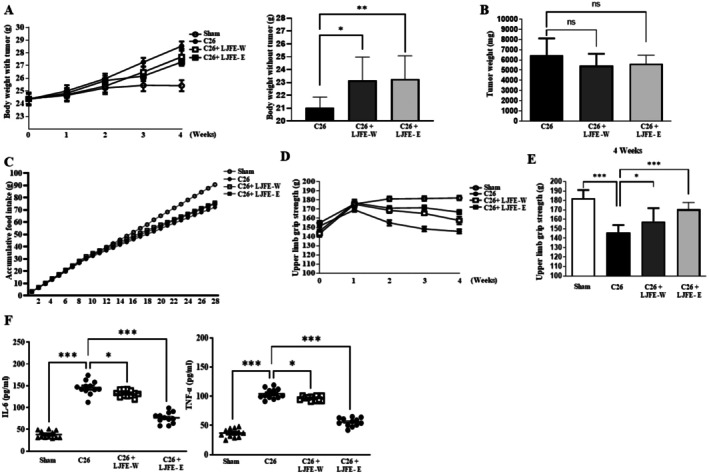
LJFE prevents body weight and grip strength in cancer cachexia mice. C26 cells were implanted subcutaneously into mice, which were then administered LJFE‐W or LJFE‐W (100 mg/kg, i.p.) for 21 days, starting on Day 7 after tumor inoculation. Healthy BALB/c and C26 mice were administered with equal volumes of saline. Body weights and tumor volumes were recorded weekly. (A) Tumor‐with body weight and tumor‐free body weight of mice. (B) Tumor weights in mice. (C) Accumulative food intake in mice. (D) Upper limb grip strength was measured from weeks 0 to 4. The tests were performed weekly and averaged across three trials. Data were expressed as mean ± SEM. (E) Grip strength of upper limb at the end of the experiment. (F) Serum levels of IL‐6 and TNF‐α in mice. Data are presented as mean ± SEM (*n* = 8). Statistical analyses were performed using one‐way analysis of variance (ANOVA). *p < 0.05, **p < 0.01, ***p < 0.001 compared to the control.

**FIGURE 6:**
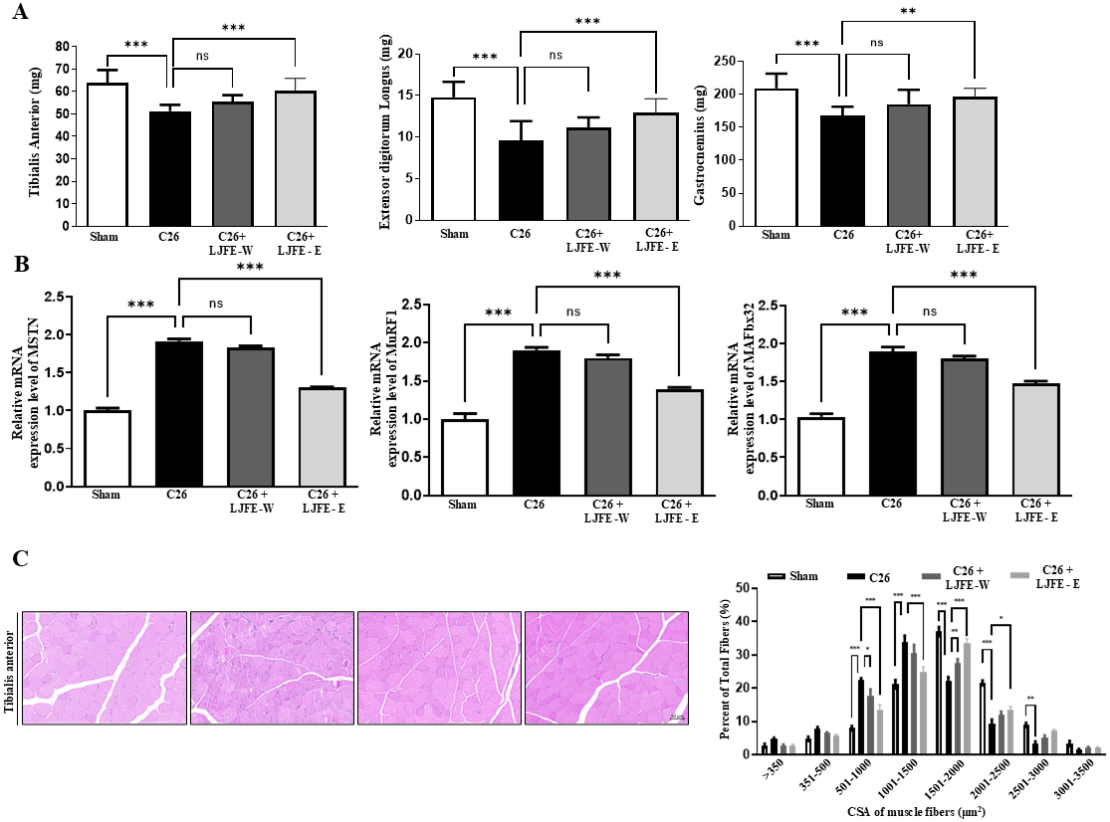
LJFE‐E attenuates muscle weight and muscle atrophy factors in cancer cachexia mice. (A) Muscle weight of tibialis anterior, extensor digitorum longus, and gastrocnemius. (B) Comparison of the relative mRNA expression of MSTN, MuRF1, and MAFbx32 in the gastrocnemius. PCR results were normalized to GAPDH. (C) Representative images of the CSA of the tibialis anterior (TA) muscle obtained by HE staining. The data show the fiber size distribution. Average fiber CSA of TA muscles. One‐way ANOVA was used to determine statistical significance. Bars represent mean ± SEM. Statistical analysis was performed using one‐way ANOVA. *p < 0.05, **p < 0.01, ***p < 0.001 compared to the control.

**FIGURE 7:**
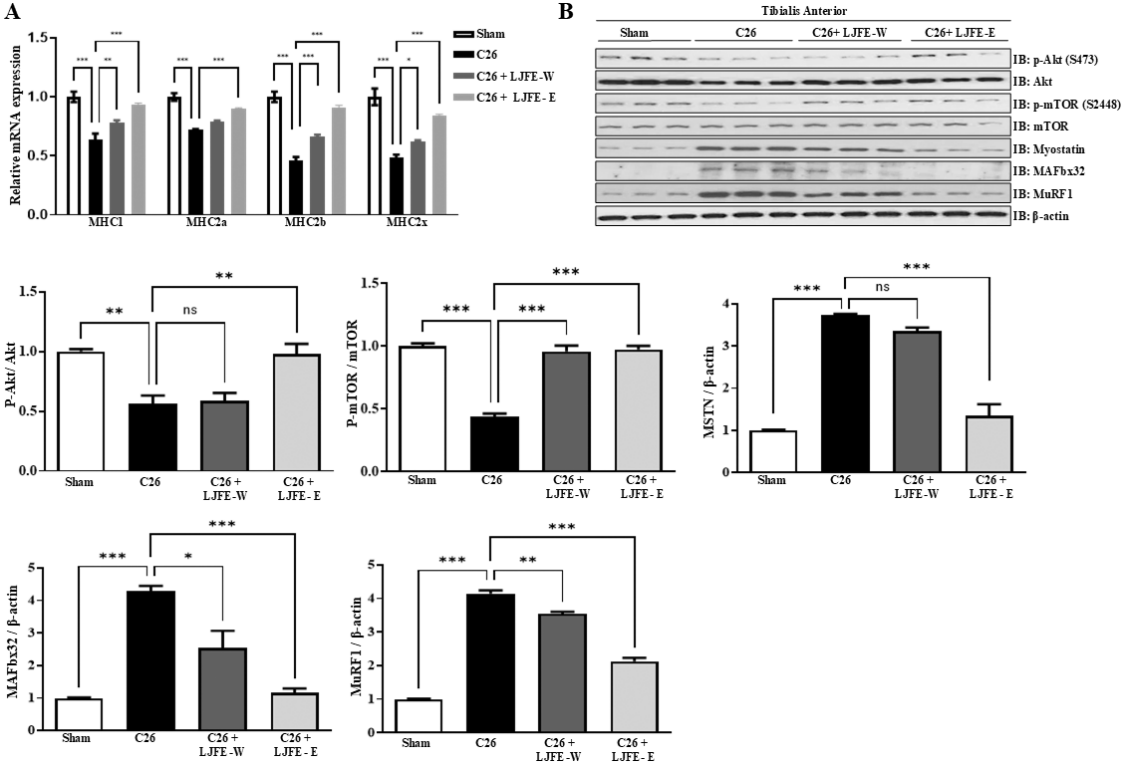
LJFE‐E attenuates muscle atrophy in cancer cachexia mice. (A) Comparison of the relative mRNA expression of MHC1, MHC2a, MHC2b, and MHC2x in the TA. PCR results were normalized to GAPDH. (B) Protein expression levels of p‐Akt, Akt, p‐mTOR, mTOR, Myostatin, MAFbx32, and MuRF1 in the TA were evaluated by western blotting. Bars represent mean ± SEM. Statistical analysis was performed using one‐way ANOVA. *p < 0.05, **p < 0.01, ***p < 0.001 compared to the control.

Throughout the experiment, the body weight of mice in the cancer cachexia group progressively increased (Figure 5A). However, at the end of the experiment, the body weight, excluding the tumor, decreased. Treatment with LJFE‐W and LJFE‐E ameliorated the decrease in tumor‐free body weight (Figure 5B). At the end of the experiment, the tumor weight was not significantly lower in the LJFE‐treated group than in the cancer‐cachexia group (Figure 5B). LJFE treatment did not increase the food intake of mice (Figure 5C). Upper limb grip strength was higher in the LJFE‐W‐ and LJFE‐E‐treated groups than that in the cancer‐cachexia group (Figure 5D,E). The serum IL‐6 and TNF‐α level in the LJFE‐treated group was significantly lower than that in the cancer cachexia group (Figure 5F). As shown in Figure 6A, the weights of the tibialis anterior, gastrocnemius, and extensor digitorum longus were significantly lower in the cancer‐cachexia group than in the sham group and restored in the LJFE‐treated group. It was confirmed that the mRNA expression of MSTN, MuRF1, and MAFbx32 in the gastrocnemius muscle decreased in the LJFE‐treated group (Figure 6B). Histological analysis revealed a reduction in the CSA of tibialis anterior muscle fibers in the cancer cachexia group, which was restored following LJFE treatment (Figure 6C). We examined MHC subtypes in the tibialis anterior and found that the expression of MHC1, MHC2a, MHC2b, and MHC2x significantly decreased in the cancer cachexia group and was restored in the LJFE‐treated group (Figure 7A). To confirm the influence of LJFE on MSTN, we examined its effects in cancer cachexia mice using western blotting. Phosphorylation of Akt and mTOR was significantly lower in the cancer cachexia group than in the sham group, while LJFE‐E treatment ameliorated the decrease in the phosphorylation of Akt and mTOR. The protein levels of MuRF1, MAFbx32, and MSTN that were increased in the cancer cachexia group were restored in the LJFE‐treated group (Figure 7B).

## 4 Discussion

The pathogenesis of cancer cachexia remains unclear. However, maintaining a balance between muscle protein anabolism and catabolism is crucial for effective treatment. Patients with colon cancer with reduced leg muscle mass exhibit stable muscle protein synthesis, a tendency for increased breakdown, and reduced postprandial protein synthesis (Williams et al. 2012). In addition, patients with cancer cachexia have been reported to show a diminished muscle protein synthesis rate relative to healthy individuals (Emery et al. 1984; Dworzak et al. 1998). Therefore, protein homeostasis remains an important therapeutic target for cancer cachexia.

Natural products, derived from living organisms such as plants, insects, and aquatic species, continue to demonstrate potential for drug development (Atanasov et al. 2021; Zhu et al. 2022). Currently, many natural product‐based drugs, such as dimethyl fumarate and podophyllotoxin analogs, are in clinical use (Talib et al. 2022). Based on these studies, this study demonstrated the alleviating effects of LJFE on muscle atrophy induced by cancer cachexia by intervening in protein metabolic imbalance.

LJFE has low cytotoxicity and potent inhibitory effects on muscle atrophy. This study focused on MSTN, a negative regulator of muscle mass.

The present study demonstrated that LJFE, its ethanol‐based extract (LJFE‐E), effectively attenuates muscle atrophy by modulating key molecular targets involved in both muscle catabolism and anabolism. In C2C12 myotubes exposed to conditioned media (CM), LJFE‐E significantly suppressed MSTN promoter activity and decreased MSTN mRNA and protein levels. Concurrently, LJFE‐E downregulated the transcription and protein expression of the E3 ubiquitin ligases MuRF1 and MAFbx32, indicating inhibition of muscle proteolytic pathways. These findings suggest that LJFE‐E interferes with MSTN‐mediated transcriptional activation of MuRF1 and MAFbx32, thereby limiting muscle degradation. This is consistent with previous studies demonstrating that MSTN inhibition results in downregulation of MuRF1 and MAFbx32 (Farhang‐Sardroodi and Wilkie 2020; Wu et al. 2024).

Furthermore, LJFE‐E treatment reversed CM‐induced suppression of Akt and mTOR phosphorylation in C2C12 myotubes, supporting its role in restoring anabolic signaling. This observation is consistent with previous reports (Chermesh et al. 2011; Hitachi et al. 2014; Choi et al. 2019), in which activation of the Akt–mTOR pathway ameliorated cancer‐induced muscle wasting and improved muscle function in preclinical models. Collectively, our findings further support the hypothesis that MSTN inhibition not only prevents proteolysis but also facilitates the reactivation of muscle protein synthesis via the Akt–mTOR signaling axis.

In addition to its effects on muscle homeostasis, the anti‐inflammatory effects of LJFE‐E may contribute to its anti‐cachectic properties. Serum concentrations of pro‐inflammatory cytokines IL‐6 and TNF‐α were markedly reduced in LJFE‐treated mice. Given that systemic inflammation is a known driver of cachexia and muscle catabolism (Wu et al. 2024), this immunomodulatory effect is likely a critical component of the observed therapeutic effects. Notably, the aqueous extract (LJFE‐W) was less effective than LJFE‐E in most parameters, implying that the bioactive components are more efficiently extracted with ethanol. This hypothesis is supported by prior studies indicating that lipophilic constituents such as hamabiwalactones and litsenolides, which possess anti‐inflammatory and antioxidant activities, are primarily ethanol‐soluble (Min et al. 2003; Koo et al. 2014).

In summary, LJFE, particularly its ethanol‐based extract, modulates both catabolic and anabolic pathways implicated in cancer‐associated muscle wasting. It exerts a multifaceted therapeutic effect by attenuating inflammation, suppressing muscle atrophy‐associated markers, and enhancing muscle protein synthesis signaling. Further studies into the pharmacokinetics, bioactive constituents, and clinical efficacy of LJFE are warranted to facilitate its potential application as a therapeutic agent for cancer cachexia.

## 5 Conclusions

This study found that LJFE prevented cancer cachexia‐induced muscle atrophy and protein imbalance and offers a promising supplement for the treatment of cancer‐related cachexia.

## Author Contributions

**Sang Woo Woo:** conceptualization (lead), data curation (lead), formal analysis (lead), investigation (equal), methodology (equal), validation (equal), visualization (lead), writing – original draft (lead), writing – review and editing (lead). **Pooreum Lim:** formal analysis (equal), investigation (equal), validation (equal). **Jihye Han:** data curation (equal), investigation (equal), validation (equal). **Ho Jin Kang:** data curation (supporting), investigation (equal). **Jae Ho Shim:** investigation (equal), writing – review and editing (supporting). **Jin Ju Lim:** validation (supporting), writing – review and editing (supporting). **Seon‐A Yoon:** investigation (supporting), resources (equal). **Hyejin Hyeon:** resources (supporting). **Young‐Min Ham:** resources (supporting). **Ji‐Gweon Park:** resources (supporting). **Yong‐Hwan Jung:** investigation (supporting). **Hyeon Soo Kim:** conceptualization (lead), funding acquisition (lead), project administration (lead), supervision (lead), validation (equal), writing – original draft (supporting), writing – review and editing (supporting).

## Conflicts of Interest

The authors declare no conflicts of interest.

## Data Availability

The data that support the findings of this study are available from the corresponding author upon reasonable request.

## References

[fsn370624-bib-0001] Amirouche, A. , A. C. Durieux , S. Banzet , et al. 2009. “Down‐Regulation of Akt/Mammalian Target of Rapamycin Signaling Pathway in Response to Myostatin Overexpression in Skeletal Muscle.” Endocrinology 150, no. 1: 286–294. 10.1210/en.2008-0959. 18801898

[fsn370624-bib-0002] Atanasov, A. G. , S. B. Zotchev , V. M. Dirsch , and C. T. Supuran . 2021. “Natural Products in Drug Discovery: Advances and Opportunities.” Nature Reviews Drug Discovery 20, no. 3: 200–216. 10.1038/s41573-020-00114-z. 33510482 PMC7841765

[fsn370624-bib-0003] Bagherniya, M. , A. Mahdavi , N. Shokri‐Mashhadi , et al. 2022. “The Beneficial Therapeutic Effects of Plant‐Derived Natural Products for the Treatment of Sarcopenia.” Journal of Cachexia, Sarcopenia and Muscle 13, no. 6: 2772–2790. 10.1002/jcsm.13057. 35961944 PMC9745475

[fsn370624-bib-0004] Bataille, S. , P. Chauveau , D. Fouque , M. Aparicio , and L. Koppe . 2021. “Myostatin and Muscle Atrophy During Chronic Kidney Disease.” Nephrology, Dialysis, Transplantation 36, no. 11: 1986–1993. 10.1093/ndt/gfaa129. 32974666

[fsn370624-bib-0005] Chermesh, I. , L. Sobotka , C. Hartman , and R. Meier . 2011. “Malnutrition and Nutrition‐Therapy: Our Neglected Responsibility.” Gastroenterology Research and Practice 2011: 842085. 10.1155/2011/842085. 21789040 PMC3140025

[fsn370624-bib-0006] Choi, D. H. , J. Yang , and Y. S. Kim . 2019. “Rapamycin Suppresses Postnatal Muscle Hypertrophy Induced by Myostatin‐Inhibition Accompanied by Transcriptional Suppression of the Akt/mTOR Pathway.” Biochemistry and Biophysics Reports 17: 182–190. 10.1016/j.bbrep.2018.12.009. 30805561 PMC6362869

[fsn370624-bib-0007] Dolan, R. D. , A. S. Almasaudi , L. B. Dieu , P. G. Horgan , S. T. McSorley , and D. C. McMillan . 2019. “The Relationship Between Computed Tomography‐Derived Body Composition, Systemic Inflammatory Response, and Survival in Patients Undergoing Surgery for Colorectal Cancer.” Journal of Cachexia, Sarcopenia and Muscle 10, no. 1: 111–122. 10.1002/jcsm.12357. 30460764 PMC6438413

[fsn370624-bib-0008] Dworzak, F. , P. Ferrari , C. Gavazzi , C. Maiorana , and F. Bozzetti . 1998. “Effects of Cachexia due to Cancer on Whole Body and Skeletal Muscle Protein Turnover.” Cancer 82, no. 1: 42–48. 9428478

[fsn370624-bib-0009] Emery, P. W. , R. H. Edwards , M. J. Rennie , R. L. Souhami , and D. Halliday . 1984. “Protein Synthesis in Muscle Measured in Vivo in Cachectic Patients With Cancer.” British Medical Journal 289, no. 6445: 584–586. 10.1136/bmj.289.6445.584. 6087973 PMC1442890

[fsn370624-bib-0010] Farhang‐Sardroodi, S. , and K. P. Wilkie . 2020. “Mathematical Model of Muscle Wasting in Cancer Cachexia.” Journal of Clinical Medicine 9, no. 7: 2029. 10.3390/jcm9072029. 32605273 PMC7409297

[fsn370624-bib-0011] Fearon, K. , F. Strasser , S. D. Anker , et al. 2011. “Definition and Classification of Cancer Cachexia: An International Consensus.” Lancet Oncology 12, no. 5: 489–495. 10.1016/s1470-2045(10)70218-7. 21296615

[fsn370624-bib-0012] Han, Y. , H. I. Kim , and J. Park . 2023. “The Role of Natural Products in the Improvement of Cancer‐Associated Cachexia.” International Journal of Molecular Sciences 24, no. 10: 8772. 10.3390/ijms24108772. 37240117 PMC10218273

[fsn370624-bib-0013] Hitachi, K. , M. Nakatani , and K. Tsuchida . 2014. “Myostatin Signaling Regulates Akt Activity via the Regulation of miR‐486 Expression.” International Journal of Biochemistry & Cell Biology 47: 93–103. 10.1016/j.biocel.2013.12.003. 24342526

[fsn370624-bib-0014] Hoek‐van den Hil, E. F. , E. M. van Schothorst , I. van der Stelt , et al. 2015. “Direct Comparison of Metabolic Health Effects of the Flavonoids Quercetin, Hesperetin, Epicatechin, Apigenin and Anthocyanins in High‐Fat‐Diet‐Fed Mice.” Genes & Nutrition 10, no. 4: 469. 10.1007/s12263-015-0469-z. 26022682 PMC4447677

[fsn370624-bib-0015] Jeong, Y. J. , I. Kim , J. H. Cho , et al. 2015. “Anti‐Osteoarthritic Effects of the Litsea Japonica Fruit in a Rat Model of Osteoarthritis Induced by Monosodium Iodoacetate.” PLoS One 10, no. 8: e0134856. 10.1371/journal.pone.0134856. 26244981 PMC4526681

[fsn370624-bib-0016] Kang, M. J. , J. W. Moon , J. O. Lee , et al. 2022. “Metformin Induces Muscle Atrophy by Transcriptional Regulation of Myostatin via HDAC6 and FoxO3a.” Journal of Cachexia, Sarcopenia and Muscle 13, no. 1: 605–620. 10.1002/jcsm.12833. 34725961 PMC8818615

[fsn370624-bib-0017] Kim, S. H. , H. J. Choi , W. K. Yang , et al. 2017. “Suppressive Effect of the n‐Hexane Extract of Litsea Japonica Fruit Flesh on Monosodium‐Iodoacetate‐Induced Osteoarthritis in Rats.” Evidence‐Based Complementary and Alternative Medicine 2017: 1791403. 10.1155/2017/1791403. 28904551 PMC5585680

[fsn370624-bib-0018] Koo, H. J. , W. J. Yoon , E. H. Sohn , et al. 2014. “The Analgesic and Anti‐Inflammatory Effects of Litsea Japonica Fruit Are Mediated via Suppression of NF‐κB and JNK/p38 MAPK Activation.” International Immunopharmacology 22, no. 1: 84–97. 10.1016/j.intimp.2014.06.007. 24968348

[fsn370624-bib-0019] Liu, D. , X. Meng , D. Wu , Z. Qiu , and H. Luo . 2019. “A Natural Isoquinoline Alkaloid With Antitumor Activity: Studies of the Biological Activities of Berberine.” Frontiers in Pharmacology 10: 9. 10.3389/fphar.2019.00009. 30837865 PMC6382680

[fsn370624-bib-0020] Min, B. S. , S. Y. Lee , J. H. Kim , et al. 2003. “Lactones From the Leaves of Litsea Japonica and Their Anti‐Complement Activity.” Journal of Natural Products 66, no. 10: 1388–1390. 10.1021/np030227i. 14575444

[fsn370624-bib-0021] Neyroud, D. , R. L. Nosacka , C. S. Callaway , et al. 2021. “FoxP1 Is a Transcriptional Repressor Associated With Cancer Cachexia That Induces Skeletal Muscle Wasting and Weakness.” Journal of Cachexia, Sarcopenia and Muscle 12, no. 2: 421–442. 10.1002/jcsm.12666. 33527776 PMC8061399

[fsn370624-bib-0022] Nikawa, T. , A. Ulla , and I. Sakakibara . 2021. “Polyphenols and Their Effects on Muscle Atrophy and Muscle Health.” Molecules 26, no. 16: 4887. 10.3390/molecules26164887. 34443483 PMC8398525

[fsn370624-bib-0023] Qu, Z. , S. Zhou , P. Li , et al. 2021. “Natural Products and Skeletal Muscle Health.” Journal of Nutritional Biochemistry 93: 108619. 10.1016/j.jnutbio.2021.108619. 33705956

[fsn370624-bib-0024] Schiaffino, S. , K. A. Dyar , S. Ciciliot , B. Blaauw , and M. Sandri . 2013. “Mechanisms Regulating Skeletal Muscle Growth and Atrophy.” FEBS Journal 280, no. 17: 4294–4314. 10.1111/febs.12253. 23517348

[fsn370624-bib-0025] Schwartsburd, P. 2019. “Cancer‐Induced Reprogramming of Host Glucose Metabolism: “Vicious Cycle” Supporting Cancer Progression.” Frontiers in Oncology 9: 218. 10.3389/fonc.2019.00218. 31019893 PMC6458235

[fsn370624-bib-0026] Setiawan, T. , I. N. Sari , Y. T. Wijaya , et al. 2023. “Cancer Cachexia: Molecular Mechanisms and Treatment Strategies.” Journal of Hematology & Oncology 16, no. 1: 54. 10.1186/s13045-023-01454-0. 37217930 PMC10204324

[fsn370624-bib-0027] Talib, W. H. , S. Daoud , A. I. Mahmod , et al. 2022. “Plants as a Source of Anticancer Agents: From Bench to Bedside.” Molecules 27, no. 15: 4818. 10.3390/molecules27154818. 35956766 PMC9369847

[fsn370624-bib-0028] Thibaut, M. M. , M. Sboarina , M. Roumain , et al. 2021. “Inflammation‐Induced Cholestasis in Cancer Cachexia.” Journal of Cachexia, Sarcopenia and Muscle 12, no. 1: 70–90. 10.1002/jcsm.12652. 33350058 PMC7890151

[fsn370624-bib-0029] Williams, J. P. , B. E. Phillips , K. Smith , et al. 2012. “Effect of Tumor Burden and Subsequent Surgical Resection on Skeletal Muscle Mass and Protein Turnover in Colorectal Cancer Patients.” American Journal of Clinical Nutrition 96, no. 5: 1064–1070. 10.3945/ajcn.112.045708. 23034966

[fsn370624-bib-0030] Wu, Q. , Z. Liu , B. Li , Y. E. Liu , and P. Wang . 2024. “Immunoregulation in Cancer‐Associated Cachexia.” Journal of Advanced Research 58: 45–62. 10.1016/j.jare.2023.04.018. 37150253 PMC10982873

[fsn370624-bib-0031] Yadav, A. , S. S. Yadav , S. Singh , and R. Dabur . 2022. “Natural Products: Potential Therapeutic Agents to Prevent Skeletal Muscle Atrophy.” European Journal of Pharmacology 925: 174995. 10.1016/j.ejphar.2022.174995. 35523319

[fsn370624-bib-0032] Yoon, W.‐J. , S. C. Kang , Y.‐M. Ham , et al. 2010. “Antioxidative and Anti‐Inflammatory Activities of Litsea Japonica Leaves.” Journal of Korean Society for Applied Biological Chemistry 53, no. 1: 27–32. 10.3839/jksabc.2010.005.

[fsn370624-bib-0033] Zhu, Y. , Z. Ouyang , H. Du , et al. 2022. “New Opportunities and Challenges of Natural Products Research: When Target Identification Meets Single‐Cell Multiomics.” Acta Pharmaceutica Sinica B 12, no. 11: 4011–4039. 10.1016/j.apsb.2022.08.022. 36386472 PMC9643300

